# It Can Be a “Very Fine Line”: Professional Footballers’ Perceptions of the Conceptual Divide Between Bullying and Banter

**DOI:** 10.3389/fpsyg.2022.838053

**Published:** 2022-02-24

**Authors:** James A. Newman, Victoria E. Warburton, Kate Russell

**Affiliations:** ^1^Sport and Physical Activity Research Centre, Health Research Institute, Sheffield Hallam University, Sheffield, United Kingdom; ^2^School of Education and Lifelong Learning, University of East Anglia, Norwich, United Kingdom

**Keywords:** bullying, banter, dividing line, professional football, interpretative phenomenological analysis

## Abstract

This study explores professional footballers’ perceptions of where banter crosses the conceptual line into bullying. The study’s focus is of importance, given the impact that abusive behaviors have been found to have on the welfare and safeguarding of English professional footballers. A phenomenological approach was adopted, which focused on the essence of the participants’ perceptions and experiences. Guided by Interpretative Phenomenological Analysis (IPA), individual semi-structured interviews (*M*_Duration_ = 44.10 min, SD = 10.81) were conducted with 18 male professional footballers (*M*_age_ = 19.83 years, SD = 2.96) from three Premier League and Championship football clubs. The findings from this study revealed several key superordinate themes in relation to the dividing line between bullying and banter. These themes included “perception,” “intentionality,” “detecting the line,” and “having a bit of banter.” The findings demonstrate how perceptions of bullying and banter are nuanced by individual differences among the players and the culture of the professional football context. Specifically, it was found that the professional football context can legitimize forms of humor blurring the lines between bullying and banter, challenging the typically positive view of the concept of banter in this environment. From an applied perspective, these findings highlight the need for coaches, players, and football clubs more broadly to address cultural expectations around banter in their environment, while educating individuals around their own perceptions of bullying and banter.

## Introduction

Recent findings of discrimination at Yorkshire County Cricket Club demonstrate serious concerns around the perceptions of what is acceptable in UK sport culture, given behaviors, such as “racial harassment and bullying,” were passed away as “friendly, good-natured banter” (BBC, 2021a). The findings in cricket echo those in professional football (or soccer), where a plethora of allegations have been linked to the safeguarding and welfare of its players (BBC, 2018, 2021b,c). More specifically, these allegations have often centered around allegations of bullying within this context (BBC, 2019, 2021b). Although research has started to respond to concerns around bullying in professional football (Newman et al., 2021a,b), it has highlighted the need for a greater understanding of the perceptions of this behavior in this context. Furthermore, given the extent to which more severe forms of banter can be normalized in professional football (Parker, 2006), it is important to explore when this behavior crosses the line into bullying.

A potential explanation for limitations in understanding around bullying, as well as banter, revolves around the conceptualization of these terms in sport. Currently, research tends to favor Olewus’ (1993, p. 8) much cited definition (Volk et al., 2014) that bullying is “an intentional, negative action which inflicts injury and discomfort on another.” Olewus’ (1993) definition also highlights an imbalance of power whereby an individual finds it difficult to defend themselves. Given football’s position as a profession, it feels noteworthy to state that workplace research echoes this view of bullying, while also outlining the persistent nature of this behavior and the inherent power differentials between the bully and victim (Sischka et al., 2021). In contrast, though significant efforts have gone into defining bullying, much less work has been invested in defining banter. To date, banter has been described as an interaction which serves to improve relationships (Dynel, 2008). Although this behavior can be aggressive, banter is seen to be challenging, yet playful, and generally occurs between friends (Steer et al., 2020). From a definitional stance, it appears that bullying and banter are clearly separate concepts. Though findings in professional football demonstrate concerns that banter may be more severe in this context, with players legitimizing various verbal and relational bullying through this term (Newman et al., 2021b). This may create ambiguity around the degree to which banter is separate from bullying. In part, this ambiguity may be reinforced by professional football’s “hidden curriculum” which teaches players they need to put up with bullying as a show of their masculine worth (Cushion and Jones, 2014). In this light, it is potentially unsurprising that welfare and safeguarding issues may be present in professional football. These issues may also be compounded by whether bullying is viewed from the victim’s or perpetrator’s perspective (Kowalski, 2000) within professional football. It is important to highlight that perpetrators, for example, often view their behaviors as more benign, humorous, and less severe than their victims.

As a response to issues in practice with understanding terms, such as bullying, researchers have sought to develop models which conceptualize this behavior. Within the sporting literature examples of such models remain relatively sparse, though Stirling’s (2009) conceptual framework of maltreatment in sport provides a guide. This model illustrates how maltreatment can be categorized into two forms: relational and non-relational, depending on whether this maltreatment occurs within the context of a “critical relationship” or not. A critical relationship is determined by whether it has significant influence over an individual’s sense of safety trust, and fulfillment of needs, with examples in sport including athletes’ relationships with their parents and coaches (Stirling, 2009). According to Stirling, bullying acts as a form of non-relational maltreatment because it occurs in the context of a “non-critical” peer-to-peer relationship, due to the bully not being in an official position of authority over the victim. This is contrasted with abuse which is the result of a “critical relationship” situation where one figure is in a position of authority, such as a coach. While this model supports our understanding of bullying in sport, subsequent research has highlighted potential issues with how some terms within the model are conceptualized. For example, in both sport and the wider workplace, bullying has been found to emerge in the context of a “critical relationship” due to the behavior of those in formal positions of power, such as coaches and supervisors (Hershcovis, 2011; Newman et al., 2021b). Moreover, Stirling’s (2009) conceptual framework was not extended to concepts, such as banter, which in its “bad” form has been found to have the same repetitive, harmful hallmarks of bullying (Steer et al., 2020). Therefore, it would appear that research may be warranted to explore this “grey area of interpretation” around bullying and banter (Steer et al., 2020), specifically in cultures which legitimize derogatory forms of banter, such as professional football (Parker, 2006).

In relation to derogatory behavior, it is worth noting that findings in sport illustrate a culture whereby abusive and bullying practices are normalized (Alexander et al., 2011; Papaefstathiou et al., 2013). Within professional football, abusive and intimidatory behaviors are commonplace (Kelly and Waddington, 2006), while bullying is often “celebrated” as a show of an individual’s masculine worth (Parker, 2006). Set within this context, it highlights the potential for welfare and safeguarding issues to occur in football. In response to this, the English Football Association (FA) sought to address these cultural issues by commissioning research around child protection (Brackenridge et al., 2004) through to the implementation of a network of Designated Safeguarding Officers (DSO; The FA, 2021). While these have been encouraging steps, the reported cases of bullying within professional football (e.g., BBC, 2019, 2021a) appear to demonstrate a preference remains to adhere to the “sport ethic,” which prioritizes performance over wellbeing (Hughes and Coakley, 1991). Furthermore, cultured beliefs in sport that performance is based on mental toughness, resilience, and perseverance (Kerr and Stirling, 2019) may also mean that player welfare around aspects, such as bullying and banter, is not considered to the extent it should be.

In addition, various limitations in sports’ safeguarding systems against bullying and more severe forms of banter appear to be evident. While important safeguarding work has been targeted at children, strategies in this area do not tend to focus on participants over the age of 18 (Rhind et al., 2015). This is problematic as allegations of bullying have been linked to under 23 team professional football players (BBC, 2019). These allegations reflect systemic issues around the safety, wellbeing, and welfare of football’s participants highlighted within the UK’s “Duty of Care in Sport” report (Grey-Thompson, 2017). Such allegations also suggest that Grey-Thompson (2017) recommendations for sports various stakeholders (e.g., coaches, parents, clubs, and national governing bodies) to care for athletes are still not being fully implemented. To compound this, research has shown even individuals who may be expected to inform, educate, and address wrongdoing, such as sport psychologists, have been found to only possess a moderate understanding of safeguarding policies (Kerr and Stirling, 2019).

Overall the findings suggest that issues around bullying and a lack of awareness around when banter becomes inappropriate may result from the organizational culture of the sport, coupled with a lack of education of the various stakeholders in this context (Owusu-Sekyere and Gervis, 2016). It is apparent that despite some initial findings from coaches around how these terms may be separated, these stakeholders play a significant role in inadvertently blurring the lines between these behaviors, shifting the borderlines around what is acceptable behavior (Kerr et al., 2016; Newman et al., 2021a). These shifts are already problematic in terms of protecting footballers’ welfare, given that banter has been found to mask discriminatory behavior, such as racism and homophobia (Adams et al., 2010; Hylton, 2018). The consequence is that this may feed a discourse among footballers where bullying and banter are used interchangeably and the conceptual divide between the two is unclear (Newman et al., 2021b). As a result, the potentially prosocial aspects of banter in sport may be lost and a more severe version of this behavior is enacted. To lose this, potentially more “inclusive” form of banter may be unfortunate as banter has been found to be central to male friendships in sport, fostering a sense of community and solidarity, while increasing cohesion and bonding (Wagstaff et al., 2017; Lawless and Magrath, 2021).

Thus, it is apparent that further work is needed to establish how professional footballers conceptualize bullying and banter and specifically the convergence and divergence in these concepts given the degree to which players discuss them interchangeably (Newman et al., 2021b). Moreover, by exploring the degree to which bullying and banter are perceived as distinct (or not), there is the potential to extend research which has shown that the gray area between these concepts leads to misinterpretation (Steer et al., 2020). Finally, given the variety of views expressed by coaches in relation to banter and how this may be distinguished from bullying (Newman et al., 2021a), it is important to explore whether players’ perceptions are equally mixed. Exploring these perceptions offers the potential to develop understanding which may safeguard players against bullying and more problematic forms of banter. Concurrently, this may also provide an opportunity to work with professional footballers to develop their critical awareness of bullying and banter in professional football to enable long-term positive behavioral change.

Therefore, due to uncertainty around how professional footballers conceptualize bullying and banter, the present study sought to explore the dividing line between these concepts. Specifically, the study aimed to explore players’ perceptions of these concepts and their views around the point at which banter crosses the line into bullying. Moreover, the present study set out to explore how bullying and banter were framed in the professional football context.

## Materials and Methods

This study was part of a larger research project which explored bullying within professional football.^1^

### Research Design

The present study adopted a qualitative, cross-sectional, and semi-structured interview design that was guided by the principles of Interpretative Phenomenological Analysis (IPA; Dwyer et al., 2019). IPA was regarded as the ideal approach to address the study’s aims, given its focus on how the person (e.g., players) makes sense of their experiences (Larkin et al., 2011) of bullying and banter in the context of professional football. Here, both the researcher and participant were engaged in a “double hermeneutic” in order to make sense of the player’s lifeworld (Dwyer et al., 2019). Furthermore, IPA was appropriate for addressing the taken-for-granted assumptions of professional football, while offering a detailed, nuanced analysis of bullying and banter (Newman et al., 2021a). By focusing on these nuances, the present study unearthed convergences and divergences within and across the participants’ accounts, maintaining the idiographic commitment of IPA (Smith et al., 2009). In addition, by exploring the conceptual divide between bullying and banter within professional football, the study was also consistent with the “contextualist” position of IPA (Larkin et al., 2006).

### Participants

Professional football was selected as the context for the present study due to the potential severity of banter, as well as celebration of bullying in this environment (Parker, 2006; Newman et al., 2021a). On this basis, it was felt that exploring the conceptual divide (e.g., the point at which one behavior is viewed as crossing into another) between bullying and banter was imperative to help safeguard the future welfare of those within football. Participants were recruited from three professional football clubs in the English Premier League and Championship divisions. In accordance with IPA guidelines (Smith, 2016), a purposive sampling strategy was utilized to identify a homogenous sample of 18 male professional footballers (*M* = 19.83, SD = 2.96, range = 18–31 years). The sample size was consistent with previous research identified as displaying good practice of IPA in sport (McDonough et al., 2011; Smith, 2016). Players were formally contracted to their club and had between 2 and 14 years of experience as a professional. Table 1 provides an overview of the participants’ demographic characteristics.

**Table 1:** Participant ages and years of experience as a professional football player.

Participant	Age	Years as a professional	Club	Division of club
James	31	14	A	Championship
Oli	21	6s	A	Championship
George	20	3	A	Championship
Charlie	19	4	B	Championship
Alfie	19	2	B	Championship
Ricky	19	2	B	Championship
Peter	19	2	B	Championship
Jamal	19	9	B	Championship
Paul	18	4	C	Premier League
Ed	18	7	C	Premier League
Dave	18	2	C	Premier League
Grant	20	5	C	Premier League
Eric	20	3	C	Premier League
Greg	20	3	B	Championship
Lenny	18	2	B	Championship
Rob	19	2	B	Championship
Kevin	21	3	B	Championship
Phil	18	2	B	Championship

### Procedure

Following institutional ethical approval, a range of potential gatekeepers were contacted to identify which English professional football clubs were willing to take part in the study. These gatekeepers were sports science and medical staff who provided support to the players but who were not responsible for their selection to the team. Once gatekeepers indicated that clubs were willing to take part, a briefing meeting was held with players who were interested in participating. After this, participants who agreed were supplied with an information sheet and completed consent forms.

The interview guide was developed and refined in accordance with best practice guidelines for IPA research within the sporting context, such that it provided a stimulus to get the participants talking, yet it was used flexibly throughout as it could not be predicted what each participant would say (Smith, 2016). Specifically, the guide was driven by the phenomenological commitment to meaning-making, with key questions being used as the basis for starting the discussion with the players (e.g., “can you tell me what banter in football is?” and “how do you recognize when it is banter rather than bullying?”), where appropriate probing techniques (e.g., “can you tell me more about that?”) were used to explicate the question (Dwyer et al., 2019). Piloting of the initial interview guide with the first three participants revealed that the questions were clear and yielded appropriate data. Therefore in accordance with previous IPA research, these interviews were included in the final analysis (Mawson et al., 2011). In order to replicate the context of the study, interviews lasted between 35 and 70 min (*M*_Duration_ = 44.11, SD = 10.81) and were conducted at the matchday venue or training ground of the participants. After the completion of the interviews, participants were reminded of how their data would be kept confidential and their rights to withdraw. Following this, all interviews were transcribed verbatim and participants’ names were replaced by pseudonyms.

### Data Analysis

In order to maintain the idiographic commitment of IPA, interviews were analyzed in turn using the guidelines set out by Smith et al. (2009). Firstly, audio files were listened back to and then, transcripts were read and re-read in order to immerse oneself in the lifeworld of the participant (Dwyer et al., 2019). The next step involved a close analysis of the text, noting exploratory comments in the right margin of the transcript. These comments were either descriptive, linguistic, or conceptual in nature, in order to identify potential meaning in the account (Smith and Osborn, 2006). Next, emergent theme titles were developed in the left margin of the text, using psychological concepts where appropriate, to capture the essential meaning in the account (Smith and Osborn, 2006). Then, emergent themes were clustered *via* a process of abstraction and subsumption which ultimately ended with a specification of superordinate themes for each case (Conroy and de Visser, 2013). This process was repeated for each participant. Finally, the combined superordinate themes from across the participants’ accounts were verified against the original transcripts, in order to ensure that the appropriate range of convergence and divergence had been captured (Conroy and de Visser, 2013). At all stages of the analysis, regular discussions were held between the authors who were all experienced in publishing IPA research. The first author completed each stage of the analysis with the other authors acting as “critical friends” (Smith and McGannon, 2018). As Smith and McGannon (2018) describe, the role of “critical friends” was not to help achieve consensus but to act as a theoretical sounding board to encourage reflection on multiple and alternative interpretations within the analysis and subsequent writing.

### Research Quality

The present study adhered to recently published guidance on achieving excellence in IPA (Nizza et al., 2021). Specifically, Nizza et al. (2021) set out four quality indicators of IPA, which the present study followed. Firstly, a “compelling, unfolding narrative was conducted” within the analysis. Here carefully interpreted extracts were selected from the participants, which told a persuasive, coherent story of how perceptual elements underpinned the conceptual divide between bullying and banter. Secondly, a “vigorous experiential account” of the participant’s extracts was developed by exploring players’ views of bullying and banter within the professional football context. Thirdly, “close analytic reading” and interpretation took place, which avoided letting quotes speak for themselves and instead inspected them for the choice of words and phrases, for their linguistic tone, use of emphasis, and for any ambiguity within them. Finally, the present study “attended to convergence and divergence” by presenting themes which showed similarities and differences between players, while also highlighting the idiosyncratic characteristics of the participants (Smith et al., 2009). The convergence and divergence are presented in the results in such a way that information on “similarities and differences and idiographical details enrich the study themes.”

## Results

Following best practice recommendations for high quality IPA studies, present study identified themes at the superordinate level. These four superordinate themes were “perception,” “intentionality,” “detecting the line,” and “having a bit of banter.” The notion of perception connects with the other themes creating a rich, cohesive narrative (Nizza et al., 2021) around how views on bullying and banter are open to interpretation. In this section, each theme is described and illustrated with quotes (Conroy and de Visser, 2013), as well as supporting interpretative commentary.

### Perception

Perception was at the heart of the individual players’ perspectives regarding whether behavior was seen as bullying or banter. In a lot of cases, footballers discussed perception from the victim’s perspective but they also highlighted how the perpetrator’s perception of their own intentions is vital. From a victim’s perspective, extracts such as James’ revealed that perception drives whether behaviors are seen as bullying, “the big thing for me is individual perception. What some people class as banter, some people class as bullying. What some people find funny, other people do not find funny.” This account highlighted the importance of an individual’s perception of their line yet showed how the placement of this varies. James’ view of the divide between bullying and banter was categorical in the sense that he used language around “some people’s classification,” as a means of clearly separating these concepts.

For younger players, such as Greg however, the divide between bullying and banter was seen as more nuanced and less clear-cut:

Oh…I dunno…it’s hard…I find it [the divide] is difficult to describe unless you gave me different scenarios, situations. Then I can probably say yeah, I think that’s bullying or no, that’s not. But I think it’s hard for me to say it because you do not know. People deal with things in different ways and there’ll be some people who’ll be happier with things being done to them or said than others. So, it’s a hard one to say.

Greg’s reference to not knowing and finding it “hard” portrayed a certain anguish and complexity with identifying these behaviors, raising questions about whether there is a line between banter and bullying. Moreover, this account echoed James’ view that these terms can be categorized. However, given Greg could not clearly distinguish the two concepts, highlighted the challenges for players to conform to professional football’s expectations regarding behavior. Latterly, his quote also implied that some individuals are regarded as being able to “take” behaviors better than others. This fueled a sense that bullying in football is a result of a potential “problem” on the victim’s side.

This problem of perception was furthered by Ed, when he discussed the differences in perspectives around bullying and banter from both the victim’s and potential perpetrator’s side:

Cos they may feel like I’m being picked on and when they speak to [the] person, they say “oh no it’s not that it’s only banter” [but] he [the perpetrator has] taken it way too far.

Ed’s extract was indicative of a feeling that speaking out around bullying behavior may be especially difficult for victims in football. Seemingly, the power to determine what is banter or not is held by the perpetrator, posing significant concerns for the welfare of other individuals. In this case, labeling this behavior as a more acceptable term of “banter” may also legitimize the bullying within the professional football culture. This was a view which Phil elaborated on:

Um…it’s tough to say. I think you have got to be the person [the perpetrator] who’s saying it to understand what they say. So, you could be sitting in the changing room and hear something come flat out of someone’s mouth and you might think to yourself “well hang on a minute I do not think that’s banter.” But to the person saying it, “I’m only joking.” I think you can only really understand whether its banter or not from the person who’s saying [it]. So, if you mean it in a certain way, you will put it across as I’m saying it that way. But you have really gotta understand, understand the person and the tone of voice and then understand well are they that type of person to say in a spiteful way and to understand whether it’s banter or not.

Phil’s view appeared to reemphasize a belief in football, particularly among the younger players in this study, that the perpetrator’s view is critical in determining whether behavior is seen as bullying or banter. This appears to warrant more education on these concepts to all involved in the game. The adoption of the perpetrator’s view also excuses this individual to some degree and takes the focus away from the importance of the victim’s perspective. It raises interesting questions about whether this is a view shaped in the academy environment which these players have recently progressed through or reflects individual maturation. Moreover, the stress placed by players, such as Phil on “needing to understand” the perpetrator, conveyed a sympathy for this individual rather than any potential victim of their behavior. This is especially problematic for any potential victims of “banter” in football, as by framing behavior this way, it creates an expectation this behavior must be accepted. Seemingly, excusing the perpetrator may be more important than safeguarding other individuals’ welfare.

Oli offered an interesting alternative view around the degree to which the perpetrator’s view may be supported, depending on insider versus outsider perspectives of banter in football:

I think on social media it would be banter, but I think people from the outside, if they have seen that. If they have seen that, they might think it’s bullying and so on.

This view was reflective of an element of seclusion in professional football (Parker and Manley, 2016) whereby the individuals within the perimeter walls or fences of the club (e.g., players and coaches) are “insiders,” whereas others interested in the sport (e.g., the media and public) are “outsiders.” Despite his status as an “insider,” Oli made references to people on the “outside” of football seeing bullying and banter in a different way, implying that players know that their behavior would not be appropriate elsewhere. Established communities of practice in professional football (Parker, 2006) appear to permit players to carry on behaving as they wish, while also allowing a more extreme version of banter and bullying. This creates a potential blindness to wrongdoing for professional football’s “insiders.” However, the advent of social media has changed the nature of professional football’s inner environment, insofar as players’ behavior can be observed by a much broader audience. Unwittingly, this creates a situation where potential wrongdoing in the form of bullying can be observed and the behavior of professional football’s “insiders” can receive greater scrutiny. Though this does highlight an important finding that safeguarding of players may only occur when wrongdoing is observable through outside channels, such as social media.

### Detecting the Line

An important perceptual element of what separated banter from bullying was the participants’ views on the point at which the line starts to be crossed between these behaviors. Many of the participants highlighted how this metaphorical line is crucial in discriminating between these concepts. Yet the concept of the “line” revealed a range of perspectives on its precise identification and whether it can even be located. Kevin’s view was reflective of this:

But I think there’s a line with banter. And some people do not know the line, some people’s lines are further away and some people’s lines are very close…You can overstep and that’s when you can see confrontations in football in the changing room.

Kevin’s various references to “the line” were symbolic of the importance placed on this hypothetical divide between banter and bullying in football, though the differences he alluded to outline the individualistic nature of perceptions of bullying.

In a similar vein, Eric highlighted the varied nature of perceptions around the dividing line between banter and bullying. As an Irish player, he illustrated something more profound around a potential passive acceptance of racism, framed as banter: “(if someone said)^2^ or something like that, another person could be like that’s racist, that’s the line for him, so that’s where you draw the line for him.” This demonstrated a worrying example of the permitting nature of sport whereby victims of potential bullying accept behaviors described as “casual racism” as part of “humorful banter” to ease racial tensions (Cleland, 2016; Hylton, 2018). Furthermore, the ways in which Eric highlighted differing perspectives around whether a racist term crossed the line or not, was indicative of an awareness within professional football that this behavior is inappropriate. Yet it also suggested that this could continue without sanctions, posing significant concerns for the welfare of players from minority ethnic groups in football.

Though Kevin and Eric discussed the “line” between banter and bullying as being quite variable, other players discussed something much more precise. Paul articulated that “once it goes to that line, there’s not a lot of width in it and it could quickly transfer to other side.” On the surface Paul’s references to there being “not a lot of width” appeared a lot clearer about when banter transitions into bullying, but on closer inspection, his extract still did not identify objectifiable means of identifying either concept in football. To this end, the players’ identification of a line felt somewhat tenuous, presenting significant challenges related to safeguarding players in football, as problem behaviors are hard to identify.

Others, though, were more categorical that this was possible:

If you noticed someone constantly picking on the same person you could realize that maybe they are taking it a step too far and if they are outright criticizing them in front of someone then you could notice it. (Rob).

In addition to this, Dave proposed that coaches may detect the line being crossed as: “Coaches would know really well by your body language, whether you are interested or not. Whether you are not having a good time or if you have [not] got loads of confidence.” In both these cases, players outlined clear behavioral information, such as repetitive criticism and observable body language, to establish bullying rather than banter.

Although the previous extracts provided some means to uncover bullying, Kevin expressed a divergent view around ease of detection using behavioral information:

Some people’s lines they do not make clear to people. And sometimes people…laugh back and really, they are not happy with the fact of what someone said but they are laughing to try and cover their insecurity. And that’s when people think that guy’s line’s not here and they take it a bit further, and it gets to a point…that’s too much and then everyone sees it in the room.

Here, Kevin’s mention of the term “insecurity” came with a connotation that those in football may pathologize wrongdoing as the victim’s problem. In this light, it is potentially unsurprising that these individuals do not “make their lines clear” or blow the whistle on wrongdoing as Kevin described. Despite this, Kevin did give the sense that the onus is still on the victim to flag these inappropriate acts. Meanwhile, this account also highlighted the fallibility of relying on behavioral cues to identify bullying as opposed to more prosocial banter in this context, as football’s participants learn to emotionally suppress negative feelings resulting from others’ behaviors. This results in a situation where it becomes “too much” as Kevin outlined and threats to individuals’ welfare become more pronounced.

### “Having a Bit of Banter”

Through their discussions around the themes of perception and the detection of the line, the players discussed the necessary yet debatable element of humor, resulting in a unanimous theme around the dividing line of “having a bit of banter.” This was characteristic of the humor deployed by players, which was largely seen as facilitative to their cohesion as a group and performance, despite it occasionally crossing the dividing line into bullying. In the main, “having a bit of banter” was articulated in relation to players’ conceptualization of banter itself:

Funny stuff, that everyone finds funny. That’s when it’s banter like if somebody said something to me and I found it funny about me. Say if someone was bantering me and I found it funny, like fair enough like, that’s banter. (Charlie).

Charlie’s account was indicative of a playful view of banter, which appears equal for both parties in the exchange, as the receiver of the joke finds the interaction “funny.” However, a deeper inspection of his account demonstrates a fragile assumption that “everyone” will find certain jokes “funny” in football. This statement conflicts earlier parts of the participants’ accounts where the individualistic nature of perception around banter and bullying was stressed. Despite players’ awareness that banter and bullying are individually experienced and perceived, it may be that professional football shapes a belief that humor is *always* ok. Jamal hinted to this, “it’s like, there’s always banter, there’s always jokes being made. But then here it’s like, everyone’s kind of cool with everyone kind of thing.” The belief that “everyone’s kind of cool, with everyone,” demonstrates a prosocial view of banter which separates it from bullying behavior, yet there are risks to this assumption given players may mask the negative sides of banter, as discussed within the detecting the line theme. Furthermore, it highlights concerns about who determines what is a joke and by what means in potentially severe contexts, such as professional football.

Nonetheless, players from other clubs, such as Eric continued the positive view of banter, suggesting that these views are grounded across football contexts, rather than at particular clubs:

Someone would be can you breathe in that? Are you ok breathing…? You know, just the clothes they are wearing, or they messed up in training or you know anything as small as that like you know.

This extract was more revealing of some of the content of this banter, which typically revolves around essential components in professional football, such as identity and performance. While Kevin agreed that this process contained positive essence, he felt it needed to be treated cautiously:

[“Having a bit of banter” it is] to try and bond with the team to try and get team cohesion about, even though that might be at one person’s expense. I think it gels the team more banter, it can be positive and healthy, it is important. But I’ve seen it can…cos it’s a very fine line; it can easily be pushed too far. So, it can be a very delicate subject.

Although Kevin continued the positive theme of banter in relation to bonding and team cohesion, the degree to which this behavior is “healthy” as he outlined could be questioned from the divergence within his own account. The precariousness around the “very fine line” he alluded to which can be easily transgressed, suggested something more troublesome for safeguarding players’ welfare. This appeared to stretch beyond one player at a particular club, given Oli’s view that “whereas banter is, can be light, it can obviously cross the line to bullying.” Oli’s language was especially noteworthy here, as while he described banter as “light” the apparent ease for this behavior to “cross the line into bullying” would suggest something different. Moreover, describing banter as “light” is reflective of a potential discourse in professional football which may downplay the severer side of this behavior. This perhaps questions more broadly the overwhelmingly positive view of banter, which is shaped by the identity required of a professional footballer.

This potential for banter to cross the dividing line into bullying was expressed more graphically by James:

(When the) word “fatty” is associated with somebody, they would never show that is affecting them because if they did then they would get it more because its classed as funny…It would be having a joke at their expense, to make them look better in front of everybody and not really caring about the effect it had on the individual.

This account provided a more sinister, severe perspective on the process of “having a bit of banter.” It once more reaffirmed the degree to which players feel the need to suppress negative feelings associated with this form of “humor.” More disturbingly, it depicted a scenario where if these feelings were revealed that this banter would become a more active form of bullying, with a blatant disregard for the welfare of its recipients. As the most experienced member of the sample, it is possible that this view was grounded in James’ longevity in the sport or may have been shaped by a different expectation for players as he came through the football system. Regardless of this though, it provided enough of a sense that the positive view of banter needed to be treated cautiously, given the degree to which others expressed that the line to bullying can be crossed.

### Intentionality

One of the most significant perceptual markers of the dividing line between bullying and banter involved intentionality. Previous research has highlighted this as a cornerstone of definitions of bullying (Olewus, 1993), including how coaches view this concept in professional football (Newman et al., 2021a). However, several contradictions were found within and between the players’ accounts here, whereby acts of bullying could be seen as accidental in nature. Furthermore, the notion of intentionality was also linked to banter behaviors. This was illustrative of something important that it is very difficult to separate concepts and the dividing line between them is blurred. Nonetheless for some players, such as Lenny, they were unequivocal that bullying was intentional:

When you know it’s affecting them. Cos if you do not know it’s affecting them then, you are still in the wrong either way but it’s difficult for you to then know, he’s not enjoying this banter and it needs to stop. But if you know it’s affecting him and you do something about it by stopping then that’s fine. But if you keep doing it and you know it’s affecting him, then that’s not right and it should not happen.

Lenny’s account separated bullying from banter based on bullying being a highly targeted act that carries clear intent despite obvious harm on behalf of the victim. It also included clear judgment about the behavior being “not right,” showing the seriousness of this bullying. Perhaps concerningly though, Lenny’s articulation of the distinction of bullying was still framed from the perpetrator’s perspective. In football, it appears that if the perpetrator thinks the behavior is not affecting the victim, then it is acceptable, rather than considering the victim’s perspective. This reinforced a troublesome sense that the professional football workplace may shape a view that perpetrators hold the power to frame potential wrongdoing as socially acceptable “banter.” This strong sense of importance placed on the combination of targeted and repetitive behaviors underpinning bullying was also reinforced by Kevin, “I think it’s consciously targeting that person…I think doing on them several, more than several times, it becomes bullying.”

The characterization of bullying as an intentional act was not common to all the players within the study. For Eric, there were contradictions with other accounts of bullying, as he described an accidental act as ignorance, “I think if there was bullying going on at a club it would be just out of ignorance I think, cos I think that person’s just like that guy’s obviously a bit like whatever.” Eric’s ignorance may not seem as severe as a targeted bullying attempt, yet it does imply that there may be a passive acceptance of bullying acts in football, rather than active attempt at challenging these behaviors. A similar contradiction was illustrated by Grant:

Obviously, they know they are gonna go deep. So, I think they know, maybe, maybe they do not know but I think most people know when they go over the line and they hold their hands up…They do not mean to do it like. There’s no wake up in the morning and thinking I’m going to bully this player, it’s just the way they are.

Both Eric’s and Grant’s attempts included a degree of uncertainty around how intentional bullying is. This was interesting, given these players were from the same club, leading to potential considerations for making sure education and welfare is delivered effectively at a local level in football. For example, Grant’s reference to “thinking they know” or “maybe they do not know” conveyed vagueness in perceptions of intentionality, though it could be questioned whether adopting this position provides some protection for the perpetrators of bullying, rather than concentrating on the welfare of the victims.

In contrast to those who clearly viewed the separation of bullying from banter to involve intentionality, Rob outlined an unintentional theme to wrongdoing.

But it’s not like you are doing it on purpose sometimes, but you are not realizing you are doing it…It might not even be intentional, it might just be how you act to that person but you do not realize how they are feeling…But I think sometimes you do not even realize you are bullying someone, cos everyone, everyone treats other people on the scale of how they can be treated.

Rob’s account further questions the centrality of intent as a component of bullying. At the same time, though it highlights the danger in assuming that banter is distinct from bullying, as individuals’ non-intentional bantering or joking on behalf of the perpetrator may be significantly impacting the recipient of this behavior in football. This problem is exacerbated by the way some players conflated bullying and banter.

Um…and just not involving them in your banter or in activities you are doing away from the club and stuff like that and if they are being victimized, they are gonna try and be somebody that they are not. Like I’ve said numerous times, it’s difficult to know when to stop the banter and the teasing and when you can have it and when you cannot. (Lenny).

Interestingly, Lenny’s combination of discussion around players not being involved in the “banter” and “being victimized” suggested something more targeted than his following point about finding it hard to know when to stop banter. These forms of ostracism and targeting sounded more like bullying, yet Lenny projected a sense, through reiterating the “numerous times” he made this point, that it is hard to determine when a joke ends and more abusive behavior begins.

This confusion between bullying and banter was maintained in other participants’ accounts:

I’d say the negatives would be, the negative would be just hurting, going out to intentionally hurt someone. Cos if your banter is doing it in spite of someone or to try and get to someone, then that’s a really bad thing. (Phil).

While Phil directly quoted the concept of banter, the process he described in terms of an intent to harm portrayed a sense that he was describing bullying. His acknowledgment that banter could be done “to try and get to someone, then that’s a really bad thing,” divulged a concerning depiction of this behavior in professional football. It hinted at a feeling that banter camouflages bullying behavior and the dividing line between these concepts may not even truly exist.

Peter continued this theme by describing a targeted process in relation to both bullying and banter adding, “um…you are picking someone out and you are going out of your way to bully them or banter them in some kind of way.” The mixing of the word bully and banter further conflated these concepts. What was evident in Peter’s eyes was that both behaviors were targeted; however, what was less clear was the degree to which he felt these concepts are distinct. Nonetheless, this account raised further concerns about the use of banter in professional football. This was supported by Oli, “probably crosses (the line) but I think like bullying, you can accidentally bully someone, ‘cos obviously the banter.” Despite attempting to define bullying, this participant showed how it can be an accidental process, which is intertwined with banter. It would appear that banter is seen by some professional footballers as a vehicle for behaviors that may drift into bullying. Overall, this suggests a darker side to the general positive view of banter in football, raising questions about the degree to which a conceptual divide with bullying exists.

## Discussion

The main purpose for the present study was to explore the dividing line between bullying and banter. Specifically, the study aimed to explore players’ perceptions of these concepts and their views around the point at which banter crosses the line into bullying. Moreover, the present study set out to explore how bullying and banter were framed in the professional football context. Within their accounts, players highlighted a range of different means by which bullying and banter may be distinguished. This included views on the perception of bullying and banter, the degree to which the line between these concepts could be detected, the process of “having a bit of banter,” and how much each concept carried an intent to harm. Nonetheless, these accounts were not consistent across participants, carrying clear implications for the safeguarding and welfare of players in professional football. On this basis, it is hoped that the findings will provide important information to professional football’s key stakeholders around managing player welfare.

Central to the participants’ accounts of the differences between bullying and banter was the importance placed on the perceptual divide between these concepts. While on the surface players described that these behaviors could be separated, the nuances within their accounts demonstrated that this is more difficult than first imagined. In relation to bullying, these findings fitted in line with previous research which has described the individualistic perception of this behavior (Thornberg and Knutsen, 2011; Thornberg et al., 2012), while extending work in this area by providing a similar conceptualization of banter. Taking these findings into account, it may provide some explanation why attempts to protect player welfare in football remain limited in their success (Parker and Manley, 2016). The individual nature of players’ perceptions of bullying and banter, and the relative lack of agency players have had in expressing their views (Pitchford et al., 2004) when codes of conducts have been designed, results in safeguarding attempts which lack efficacy.

The lack of success of safeguarding approaches in professional football may also be partly explained by a consistent finding across the participants’ accounts that the perpetrator frames the decision around what bullying and banter are in this context. Players expressed potentially misguided views around needing to understand the perspective of the perpetrator, giving rise to a sense that perceived bullying is the victim’s “problem.” For example, players expressed the view that if the perpetrator did not mean harm as part of their humor (Kowalski, 2000), then this must be viewed as banter. This revealed concerns that for some players, they may not recognize that banter can be offensive and cross the line of acceptability (Steer et al., 2020) and also raised doubts around the extent to which they would reflect on their potentially inappropriate actions. The results are exclusionary forms of banter which “cross this line” (Lawless and Magrath, 2021) being masked in professional football. Here, players seemingly appear to accept and reproduce a disciplinary form of humor (Edwards and Jones, 2018) which previous research suggests (Parker, 2006) they may have observed from their coaches.

Perceptions around inclusionary and exclusionary forms of “banter” also linked to how participants determined the line between bullying and banter. Worryingly, players in some cases appeared to suggest that casual racism may even be accepted in some cases (Cleland, 2016; Hylton, 2018) suggesting a more extreme form of banter may be acceptable in professional football. This even contrasts with other masculine sporting contexts, such as cricket, where racism is seen to transgress acceptable forms of banter (Lawless and Magrath, 2021). It would appear that as part of professional football’s established community of practice (Parker, 2006), players learn that diversity almost acts as an excuse for bullying behavior to be disguised as banter. In turn, the word banter legitimizes these discriminatory behaviors as socially “acceptable” in the professional football context. Furthermore, in comparison with findings with professional football coaches who highlighted discrimination as clearly identifying bullying in football (Newman et al., 2021a), the present study shows that for players, the dividing line between bullying and banter may be shifted in a more severe direction.

The more severely positioned divide players articulated may go some way to explaining why welfare concerns exist in football around the use of peer-group “banter,” which may otherwise be interpreted as bullying (Oliver and Parker, 2019). Indeed, this idea of a dividing line itself may allow players to protect themselves from being accused of inappropriate banter, so long they stay within the perceived territory of what professional football deems “acceptable” behavior. In this light, it is understandable why participants highlighted that detecting the line between banter and bullying may be difficult as victims learn to “laugh off” inappropriate actions toward them. Consistent with findings with coaches (Newman et al., 2021a), the need to conform to a masculine identity within professional football leads players to feel the need to “perform” a masculine identity (Connell, 2008). This results in them hiding forms of banter which they have found unacceptable.

Given players may hide the negative effects of banter, there was also a concerning assumption in some of their accounts that it would be observable when behavior crossed the line between banter and bullying. Given previous research in football has shown that victims of wrongdoing may not display signs that it is happening (Newman et al., 2021b), players may not be in the best position to detect lines between more appropriate forms of banter and bullying. Likewise, other players felt coaches may be in a good position to identify these behaviors instead. Though once more, this belief may be problematic, as coaches have been found to be susceptible to blurring the lines between bullying and banter and may overestimate their ability in addressing these types of behaviors (Baar and Wubbels, 2013; Newman et al., 2021a).

Although the conceptual divide between bullying and banter may be difficult to distinguish at times, players did identify a more prosocial form of banter. In line with previous research, banter can fulfill an important role in creating camaraderie (Kennedy, 2000) among male footballers, while at the same time, players in the present study highlighted the positive impact this has on team cohesion. As such banter in this form offers the potential to aid bonding and ultimately performance in football, in a similar fashion to other sports, such as Rugby Union (Wagstaff et al., 2017). Therefore, it would appear that banter in professional football is not necessarily a negative act, akin to bullying and instead can be seen as a playful, jocular interaction which unites friendship (Steer et al., 2020).

It should be noted though that despite the more positively framed view of banter, within the “having a bit of banter” theme, players offered cautionary points about the potential for this humor to quickly cross the line into bullying. Thus, the potential warning signs around when this line of acceptability is being approached appear not to be observable to players. They highlighted examples, such as how a focus on individual appearance, can lead to a process of “banter” which would target an individual regardless of their feelings. From a contextual stance, it highlighted the need for individuals to achieve a particular identity in football remains (Parker, 2006) and if players do not achieve this they can expect to receive greater levels of derogation. From a theoretical stance, it would appear that this may drive a process of negative downward social comparison (Wills, 1981), through the use of banter, when players do not conform to these ideals. This carries a worrying implication for the welfare of players from a self-presentation perspective (Leary and Kowalski, 1990). Here, there is the potential for individuals to become preoccupied by concerns around managing their impression and leading them to carry a strong protective motivation to avoid being seen as different. This may have a significant bearing on their overall sense of self and wellbeing.

The potential harmful impact of the often positively view of banter linked to the final theme expressed around intentionality. In line with previous conceptualizations within both the mainstream psychological literature (Olewus, 1993), as well as in football specifically (Newman et al., 2021a), this marked a clear differentiation of bullying from banter for some participants. In other cases, bullying and banter were both framed as intentional acts which set out to hurt individuals or exclude them from the team, further blurring the conceptual divide between them. From a contextual standpoint, this can be understood through a process of “situated learning” in professional football, where players learn how to behave as part of the sport’s culture (Parker, 2006). Utilizing the lens of this conceptual model of learning (Lave and Wenger, 1991), players in this study may have socially learned within football that banter may need to be more targeted than in other domains. This appears to provide support for the notion in professional football that for individuals to achieve peer-group credibility, they need to give insults often framed in the form of banter, to the point at where the recipient snaps (Parker, 2006). The result is a form of “bad” banter which manifests itself in professional football.

Finally, the more “accidental” form of bullying described by some players further blurs the conceptual line with banter. This mirrors other findings in sport that argue perpetrators do not intentionally carry out hurtful actions, which nonetheless are viewed as bullying (Kerr et al., 2016). As such these findings challenge previous definitions of bullying (e.g., Olewus, 1993; Volk et al., 2014), which have highlighted the importance of a hostile form of intent in identifying this behavior. Sport and football specifically may be unique in this regard, in normalizing and potentially celebrating bullying behaviors (Parker, 2006; Kerr et al., 2016), meaning this harmful intent is much more difficult to discern and may occur by accident. Moreover, by viewing these behaviors as accidental, it may indirectly legitimize players to continue using them, creating concerns that serious wrongdoing may be challenged or addressed. In terms of the safeguarding of welfare of individuals in these contexts, this presents a worrying picture around conceptual ambiguity and the normalization of inappropriate behaviors in football and wider sport.

Overall, the present study’s findings provide an important conceptual and contextual addition to the research literature on bullying and banter. Given the variety in perceptions around bullying and banter, it highlights a blurred line between these concepts. This adds evidence to claims (Kerr et al., 2016) that classifying behaviors as bullying and banter based on strict definitional criteria may be less useful in professional football. Instead, the focus should be on the behaviors enacted by individuals within this environment, as well as their perceptions of how these behaviors impact their wellbeing (Kerr et al., 2016). The findings in relation to banter in sport specifically appear to fit with this viewpoint as participants construed this behavior in many ways. In line with the theoretical propositions of Benign Moral Violation theory (McGraw and Warren, 2010), players outlined how this banter can be offensive yet also occurs in a situation among friends within a team. Thus, the present findings added further weight to claims banter is a complex and contradictory phenomenon in sport (Lawless and Magrath, 2021).

From a contextual standpoint, the present study also highlights the importance of sport and particularly football, in framing views of bullying and banter. Due to the tendency of players to frame both behaviors on the peer-to-peer level, the findings extend Stirling’s (2009) conceptual model of maltreatment in sport by suggesting that banter also occurs as part of a “non-critical” relationship in the same way as bullying. The present findings also tend to reaffirm that bullying (and banter) occurs in sport within relationships where there is a power imbalance but the perpetrator is not in a position of authority (Stirling, 2009). This may make the detection of this behavior challenging, as the players highlighted bullying occurs through the social and emotional means (e.g., excluding other players and excessive banter) proposed by Stirling (2009), rather than through overt physical actions. Moreover, the findings give credence to the persistence of the “sport ethic” (Hughes and Coakley, 1991) in professional football which focuses less on player wellbeing and potentially more on performance. The degree to which players appeared to legitimize more severe forms of banter, as well as the degree to which the perpetrator’s view on what may or may not be acceptable behavior is upheld, still presents significant issues in this context. Ultimately, this might explain how and why reporting wrongdoing through safeguarding channels may remain difficult, posing continued concerns for welfare in football.

### Applied Implications

As a result of the findings within the present study around how the participants conceptualized the dividing line between bullying and banter, two implications are set forward. Firstly, football’s key stakeholders (e.g., coaches, players, sporting directors, and shareholders) need to be educated around the blurred conceptual line between bullying and banter, as well as the subsequent impact this may have on individual welfare. Specifically, education needs to realize the fluid, rather than binary nature of banter in professional football (Lawless and Magrath, 2021). This fluidity means that individuals need to realize at what point the line between banter and bullying might start to be approached, as banter can quickly cross the line from acceptable, inclusionary forms of this behavior to unacceptable, exclusionary actions which mimic bullying. Education programs in professional football need to reaffirm that exclusionary forms of banter cannot be legitimized within this sport, as they transgress “acceptable” behavior (Lawless and Magrath, 2021). Similarly, more effort is needed to identify “loaded” forms of banter with professional football’s stakeholders, given harmful comments are often knowingly masked as being inoffensive. Secondly, linked to the previous point, perceptions of bullying and banter need to be challenged at all levels of professional football. Interventions need to address the normalization of severe behaviors and “banter” in this environment and provide clear channels for individuals to be able to speak out about their concerns. More work needs to focus on the actual behavior of football’s various stakeholders, challenging the sense that the acceptability of actions is framed from the perpetrator’s perspective. This needs to target individual, club and wider organizational level perceptions of bullying and banter, to proactively manage wellbeing in this context. For example, work focused on academy contexts may be useful to create a different culture around these concepts for new players as they enter and develop through professional football.

### Limitations and Future Research Directions

Although the study made an important contribution to further understanding the conceptual divide between bullying and banter, it does present limitations that need consideration. Firstly, while the present study addressed an important issue by exploring players’ perceptions of the divide between bullying and banter, there is still a need to engage other stakeholders’ perspectives of these concepts, to better safeguard individuals in football. A focus on the views of individuals who are employed to protect wellbeing in football, such as safeguarding leads, player care officers and sport psychologists may be particularly useful in this regard. Secondly, although the present study has identified important information about the often-blurred conceptual divide between bullying and banter, it did not focus specifically on the outcomes of these behaviors. Future research may seek to explore the outcomes for both perpetrators and victims of bullying and banter in sport, to understand the impact more fully on wellbeing. Thirdly, the present study may present linguistic issues which may be worth consideration. The use of the concepts bullying and banter was relevant to UK professional footballers, but it is less known whether these concepts are applicable within other languages or other versions of the English language. Therefore, future studies may explore the relevance of these terms both within and outside of professional football to explore whether there are similar issues in distinguishing between them. Finally, the present study remained limited to the perspective of male professional footballers. Future studies may engage the perspectives of other players, such as women professionals and male and female grassroots participants, to explore whether the findings are systemic across football as a sport.

## Conclusion

The present study makes an important contribution to the literature on bullying and banter in various ways. Firstly, we identified the often-blurred conceptual divide between bullying and banter. This serves to challenge potential misconceptions around banter being seen as a solely prosocial behavior in football. Secondly, we unearthed the importance of individual perceptions in determining what appropriate behavior is. This provides important information around the need to focus on these perceptions and avoid binary classifications of bullying and banter. Finally, we identified the importance of the culture of professional football in shaping perceptions of these behaviors. It is hoped that the present findings provide important information which can educate those in sport around the concepts of bullying and banter, while at the same time informing the future development of safeguarding and welfare programs.

## Data Availability Statement

The datasets presented in this article are not readily available because of the nature of this research, participants of this study did not agree for their data to be shared publicly, so supporting data is not available. Requests to access the datasets should be directed to JN, j.newman@shu.ac.uk.

## Ethics Statement

The studies involving human participants were reviewed and approved by University of East Anglia, School of Education and Lifelong Learning Research Ethics Committee. The participants provided their written informed consent to participate in this study.

## Author Contributions

JN prepared the original draft of the manuscript and led the study’s administration and investigation. VW and KR supervised, reviewed, and edited this work. All authors listed have made a substantial, direct, and intellectual contribution to the work and approved it for publication.

## Conflict of Interest

The authors declare that the research was conducted in the absence of any commercial or financial relationships that could be construed as a potential conflict of interest.

## Publisher’s Note

All claims expressed in this article are solely those of the authors and do not necessarily represent those of their affiliated organizations, or those of the publisher, the editors and the reviewers. Any product that may be evaluated in this article, or claim that may be made by its manufacturer, is not guaranteed or endorsed by the publisher.

## Footnotes

^1^To date, a previous research article focusing on conceptualizing bullying in football has been published from this research project (Newman et al., 2021b). Conceptualizing bullying in adult professional football: A phenomenological exploration, *Psychology of Sport and Exercise*, 101883. doi: 10.1016/j.psychsport.2021.101883 a further paper is under review. The data presented in the present study are unique from this previously published/submitted research, as is the focus of this work.

^2^The term used by the participant is a racial slur referring to people who are from the Traveller community.
